# Coping Strategies and Complicated Grief in a Substance Use Disorder Sample

**DOI:** 10.3389/fpsyg.2020.624065

**Published:** 2021-01-15

**Authors:** Beatriz Caparrós, Laura Masferrer

**Affiliations:** ^1^Department of Psychology, University of Girona (UdG), Girona, Spain; ^2^CAS Girona, Mental Health and Addiction Research Group, Institut d’Assistència Sanitària (IAS), Institut d’Investigació Biomèdica de Girona (IDIBGI), Girona, Spain

**Keywords:** coping strategies, complicated grief, substance use disorder, alcohol, cocaine, heroin

## Abstract

**Background**: Previous research has identified a link between the loss of a significant person, grief complications, and substance abuse. People with substance use disorder (SUD) are more vulnerable to complicated grieving symptoms following loss. From sociocognitive theories, the model of coping with stress assumes that substance use is one of the responses used to cope with traumatic life events. The main objective of this study is to identify the coping strategies of people with SUD and to analyze their relationship to complicated grief (CG).

**Methods**: A sample of 196 bereaved drug-dependent patients was assessed, after providing written consent, in sociodemographic variables, drug and bereavement related characteristics, CG symptomatology (Inventory of Complicated Grief) and coping strategies (Coping Strategies Inventory).

**Results**: There are differences in relation to the coping strategies used among patients with CG, using more those focused on emotional expression, social withdrawal, wishful thinking, and self-criticism.

**Conclusion**: We can conclude that, in general, CG in patients with SUD is more associated with the use of less adaptive coping strategies. This data can contribute to a better understanding of the different variables involved in the grieving process among people with SUD. It is important to point out the clinical implications of addressing what the coping strategies associated with improved grief outcomes among people with addiction problems are.

## Introduction

The identification of risk factors and protective factors associated with substance use and concurrent problems is a substantial area of research in terms of preventive and intervention strategies in vulnerable populations, such as people with substance use disorders (SUD). Numerous studies have investigated the relationship between coping and different clinical disorders course (Taylor and Stanton, 2007; Al-Gamal et al., 2016), and have considered coping strategies as an important variable for a better understanding of addiction problems (Walker and Stephens, 2014). Coping has been broadly defined as “cognitive and behavioral efforts to manage specific external or internal demands (and conflicts between them) that are appraised as taxing or exceeding the resources of a person” (Lazarus, 1991, p. 112). In addition, Skinner and Zimmer-Gembeck (2016) described coping strategies as a basic process integral to adaptation and survival, depicting how people detect, appraise, deal with and learn from stressful encounters.

Previous research has identified a link between the loss of a significant person, grief complications and substance abuse (Denny and Lee, 1984; Zuckoff et al., 2006). In addition, people with substance use disorders (SUD) often report personal losses in their life histories that make recovery difficult (Furr et al., 2015), and are more vulnerable to report complications in bereavement after the loss of a significant individual (Masferrer et al., 2017). Complicated grief (CG) has been defined as a deviation from the normal (in cultural and societal terms) grief experience in either time course, intensity, or both, entailing a chronic and more intense emotional experience, which either lacks the usual symptoms or in which the onset of symptoms is delayed (Stroebe et al., 2007).

Estimating the incidence of CG symptoms is not easy since its study has been carried out on different samples and with different definition criteria (Stroebe et al., 2007). In general, population studies prevalence ranges from 2.4 to 22.7% (Bonanno, 2006; Fujisawa et al., 2010; Kersting et al., 2011; Newson et al., 2011; Wittouck et al., 2011). In clinical samples, research prevalence vary from 18.6% among hospitalized patients with unipolar depression (Kersting et al., 2009) to between 20 and 34% among psychiatric patients (Zisook et al., 1985; Zisook and Lyons, 1990; Prigerson et al., 2002), 24.3% among bipolar disorder patients (Simon et al., 2005), 33.3% among a mixed sample of psychiatric outpatients (Piper et al., 2001), and 34.2% in SUD patients (Masferrer et al., 2017).

From sociocognitive theories, the model of coping with stress assumes that substance use is one of the responses used to cope with traumatic life events (Wills and Hirky, 1996). In general, research data in the field of addictions suggest that problem-centered strategies are related to a more positive evolution while avoidance strategies are related to a worse evolution (Feil and Hasking, 2008). Longitudinal and cross-sectional studies conducted in both community samples (Johnson and Pandina, 2000) and clinical samples (Hasking and Oei, 2002; Feil and Hasking, 2008; Adan et al., 2017) show that both avoidance and emotion-focused coping strategies are proper indicators of alcohol use (Coriale et al., 2012). In relation to opioid use, mixed results were found in terms of coping, with some studies stating that behavioral coping was more widely used than cognitive coping strategies, or a report of less use of problem-focused coping and less use of emotion-focused coping in an abstinent period (Pal and Chavan, 1996; Hyman et al., 2009).

Most people encounter a challenge in their emotional states as a component of regular day to day existence. Particularly, the majority of SUD individuals may experience overpowering feelings, regardless of whether as a consequence of the impact of drugs, withdrawal, or the everyday relation with stressors. Indeed, figuring out how to balance these emotional states is a core aspect related to SUD treatment (Tariq and Jameel, 2020).

Sometimes, when coping strategies are deficient, bereaved people can easily develop complications in bereavement, which is associated with multiple physical and mental problems that cause social disruption and significantly reduce quality of life (Latham and Prigerson, 2004; Boelen and Prigerson, 2007; Stroebe et al., 2007; Masferrer et al., 2017). Previous research on coping and CG has found that, in older adults who had lost their spouse, engagement and rumination were associated with a worse prognosis of grief (Prigerson et al., 1995a); in samples of young college students, avoidant emotional coping was also found to be associated with increased severity of CG (Schnider et al., 2007). Overall, results show that emotion-focused coping is associated with poorer mental health outcomes (Coyne and Racioppo, 2000).

Although there is a lot of research that has analyzed the relationship between coping and substance use (Anderson et al., 2006), and also research that has studied the relationship between grief and coping strategies (Schnider et al., 2007), there are no studies, to our knowledge, that have analyzed coping strategies in such a specific population as people with substance use disorder with associated CG. The main objective of the present study was to identify the coping strategies of people with SUD and to analyze their relationship to CG. The specific objectives were (1) to study the relationship between CG and its factors, and the different coping strategies; (2) to analyze if there are differences between the CG group and adaptive grief group regarding different types of SUD and the coping strategies used and, finally, and (3) to identify the significant coping strategies in predicting CG in SUD patients.

## Methodology

The current study is part of a larger research project that aims to investigate different variables related to CG in SUD patients (Masferrer et al., 2015). The sample size was calculated based on the estimated prevalence of CG in the general population and with an alpha level of 0.05 and an accuracy of 0.05. According to previous studies (Lobb et al., 2010) we assume a 15% CG prevalence. The prevalence of CG in this SUD sample was 34.2%.

### Participants

The sample was based on a consecutive non-probabilistic convenience sample of 196 SUD patients at the Public Addiction Treatment Centre in Spain. The inclusion criteria were that they: (1) had a diagnosis according to Diagnostic and Statistical Manual of Mental Disorders (DSM-IV-TR) criteria of alcohol, cocaine or heroin dependence; (2) had suffered the loss of a significant person at some point in their life and before the year prior to the study, and (3) abstinence during the last month. Related to the sociodemographic variables, 78.1% were men and 20.2% women. The average age was 45.6 years (sd = 10.14, min = 22, max = 74). 37.2% were married or had a partner. More than a half of the sample (65.3%) reported primary education and the rest had secondary or higher education. Regarding the main diagnosis, 68.9% had alcohol dependency, 18.4% heroin dependency, and 12.8% cocaine dependency. Regarding professional situation at the time of the evaluation, 63.3% reported being retired, unemployed, inactive or receiving disability aid, and 36.7% were actively working. Mean time since the death of the significant person was 11.97 years (sd = 11.07, min = 1, max = 46). Complicated grievers reported less time since death (9.36 years; sd = 9.78) than adaptive grievers (13.33 years; sd = 11.48; *p* = 0.02).

### Measures

Characteristics related to gender, age, marital status, educational level, and professional situation were evaluated through an *ad-hoc* questionnaire (for more information see Masferrer et al., 2017).

The Spanish version of the Inventory of Complicated Grief (ICG; Prigerson et al., 1995b; Limonero et al., 2009) was used, which provides a total score for CG and three factors (memories of the deceased, sensation of emptiness, and experience of the deceased) through 19 items with a five-point Likert scale. The maximum score is 76 and the cut-off point used was 25 according to the original version. The internal consistency of the Spanish version was 0.88 and the test-retest reliability was 0.81.

The Coping Strategies Inventory Spanish version (Tobin et al., 1989; Cano et al., 2006) was used to assess coping. This instrument consists of 40 items on a Likert scale (0: nothing ‐ 4: totally agree) and evaluates eight primary scales: (1) Problem Solving (PS), (2) Cognitive Restructuring (CR), (3) Social Support (SS), (4) Emotional Expression (EE), (5) Problem Avoidance (PA), (6) Wishful Thinking (WT), (7) Social Withdrawal (SW), and (8) Self-Criticism (SC). There are four secondary scales: Problem Focused Engagement (composed of problem solving and cognitive restructuring), Emotion Focused Engagement (Social Support and Express Emotions), Problem Focused Disengagement (Problem Avoidance and Wishful Thinking), and Emotion Focused Disengagement (Social Withdrawal and Self Criticism), and two tertiary scales: Engagement (Problem and Emotion Focused Engagement) and Disengagement (Problem and Emotion Focused Disengagement). Cronbach’s alpha of the Spanish version ranged from 0.70 to 0.86 with the average being 0.81.

### Procedure

The psychometric tests were administered following the Organic Law 15/1999 regarding the Protection of Personal Data. Informed consent was obtained from all participants and the protocol was approved by the Institutional Ethics and Research Review Board of the Institut d’Assistència Sanitària (IAS). According to them, the study expires with the current regulations of the International Conference of Harmonization of the Procedure of Good Clinical Practice (CPMP/ICH/135/95) as well as the International Guidelines for the Ethical Review of Epidemiological Studies. Those who voluntarily wanted to participate and signed the informed consent were evaluated through a clinical interview with the psychologist (second author) and were administered the psychometric questionnaires included in the protocol. The duration of each of the interviews carried out was about 1.5 h.

### Statistical Analysis

The CG group was formed by the participants scoring higher than 25 on the ICG (*n* = 67). The alcohol group and the heroin and cocaine group were formed according to the main diagnosis. We use these two groups in order to separate legal and illegal substances group. Pearson correlations were used to test associations among quantitative variables of coping and CG. The Student’s *t*-test was used to determine differences between groups (main substance diagnoses/CG) in coping. In order to identify the coping strategies related to the presence of CG, we conducted a multiple linear regression model. Data processing and analysis were performed using the SPSS statistical program version 21.0 for Windows (IBM Corp., Armonk, NY, United States).

## Results

### Coping Strategies and Grief Symptoms

Associations between the total ICG scale, its factors and coping strategies were analyzed (Table 1). Preliminary analyses in the total sample showed that desiderative thinking and social withdrawal correlated positively with the total CGI score and with all its factors. Express emotions were also positively associated with ICG except for the Presence experience of the deceased factor. Self-critical coping, although of lesser magnitude, was also positively and significantly associated with ICG and all its factors, except the empty feelings factor. With this same factor, the subscale of problem avoidance also correlated positively. Social withdrawal obtained the highest rates. The second order scales, Problem Focused Disengagement and Emotion Focused Disengagement, correlated positively and significantly with all the scores of CG. Although it is important to note that the second and third order factors related to disengagement coping showed stronger correlations than those of engagement.

**Table 1:** Correlations among complicated grief and coping strategies.

Coping CG	PS	CR	SS	EE	PA	WT	SW	SC	PFE	EFE	PFD	EFD	E	D
Total CGI	0.120	0.085	0.049	0.204^**^	0.130	0.229^**^	0.315^**^	0.155^*^	0.124	0.159^*^	0.258^**^	0.274^**^	0.166^*^	0.313^**^
MD	0.086	0.064	0.045	0.194^**^	0.125	0.222^**^	0.294^**^	0.154^*^	0.091	0.151^*^	0.250^**^	0.263^**^	0.143^*^	0.301^**^
EF	0.099	0.068	0.030	0.186^**^	0.166^*^	0.201^**^	0.274^**^	0.102	0.101	0.136	0.263^**^	0.216^**^	0.140	0.275^**^
PED	0.118	0.137	0.008	0.133	0.066	0.188^**^	0.320^**^	0.197^**^	0.156^*^	0.090	0.183^*^	0.307^**^	0.141^*^	0.300^**^

Complicated grief (CG): Total CGI, total score complicated grief inventory; MD, memories of the deceased; EF, empty feelings; PED, presence experience of the deceased. Coping strategies: PS, problem solving; CR, cognitive restructuring; SS, social support, EE, express emotions; PA, problem avoidance; WT, wishful thinking; SW, social withdrawal; SC, self-criticism; PFE, problem focused engagement; EFE, emotion focused engagement; PFD, problem focused disengagement, EFD, emotion focused disengagement; E, engagement (problem and emotion); D, disengagement (problem and emotion).

* Correlation is significant in the level 0.05.

** Correlation is significant in the level 0.01.

### Differences Between Adaptive Grievers and Complicated Grievers Groups

Differences in coping between groups of SUD patients with and without CG are analyzed (Table 2). The CG group score significantly higher on self-criticism, express emotions, wishful thinking, and social withdrawal. In same direction, significative differences were also shown on the second‐ and third-order scales in both problem-focused and emotion-focused disengagement.

**Table 2:** Differences between adaptive grievers and complicated grievers groups.

	Adaptive grief (*N* = 129)	Complicated grief (*N* = 67)			
	M (SD)	M (SD)	Cohens’ d	*t*	*p*
**Coping strategies**					
Problem solving	14.66 (5,34)	15.75 (4.33)	0.22	−1.54	0.152
Self-criticism	8.57 (7.07)	10.91 (7.02)	0.33	−2.21	0.028
Express emotions	8.77 (6.01)	11.10 (6.05)	0.39	−2.58	0.011
Wishful thinking	13.71 (5.59)	15.81 (3.81)	0.44	−2.75	0.002
Social support	8.96 (5.63)	9.46 (5.54)	0.09	−0.60	0.553
Cognitive restructuring	9.38 (5.40)	9.55 (5.42)	0.03	−0.21	0.883
problem avoidance	5.36 (4.49)	6.34 (5.66)	0.19	−1.23	0.222
Social withdrawal	7.16 (5.07)	10.19 (5.48)	0.57	−3.87	0.001
**Second factor scales**					
Problem focused engagement	21.04 (8.90)	25.30 (7.87)	−0.15	−0.98	0.330
Emotion focused engagement	17.73 (9.62)	20.57 (9.22)	−0.30	−1.97	0.048
Problem focused disengagement	19.08 (6.87)	22.15 (6.93)	−0.45	−2.96	0.003
Emotion focused disengagement	15.72 (9.99)	21.10 (9.78)	−0.54	−3.60	<0.001
**Third factor scales**					
Engagement	41.77 (15.55)	45.87 (15.20)	−0.27	−1.76	0.078
Disengagement	34.80 (14.17)	43.25 (14.35)	−0.59	−3.95	<0.001

In relation to the types of substance, there are no significant differences (*t* = −1.07, *p* = 2.84) in the CG scores between the alcohol group (M = 20.87, SD = 16.84) and the heroin and cocaine group (M = 23.64, SD = 16.46). In the first group, there was 30% of the cases with CG, and in the second group was 40% of CG.

Table 3 shows that significant differences exist between the alcohol group and the heroin and cocaine group in wishful thinking, cognitive restructuring, and problem avoidance, with the patients with alcohol use showing higher scores in all of them, although with moderate effect sizes. In the second order factors, the existing differences are found in relation to the focus of the problem, obtaining higher scores in the alcohol group in both engagement and disengagement. It is also this group that obtains higher and significantly different scores in the third order scales problem and emotion focused engagement.

**Table 3:** Differences in coping between alcohol dependence group and heroin and cocaine group main diagnosis.

	Alcohol group (*N* = 135)	Heroin and cocaine (*N* = 61)			
	M (SD)	M (SD)	Cohens’ d	*t*	*p*
**Coping strategies**					
Problem solving	15.41 (4.71)	14.20 (5.65)	0.23	1.57	0.119
Self-criticism	9.33 (7.26)	9.46 (6.86)	−0.02	−0.12	0.904
Express emotions	10.07 (5.98)	8.44 (6.28)	0.27	1.74	0.083
Wishful thinking	15.09 (4.79)	12.97 (5.60)	0.41	2.72	0.007
Social support	9.44 (5.62)	8.46 (5.49)	0.18	1.14	0.258
Cognitive restructuring	10.02 (5.63)	8.15 (4.61)	0.36	2.46	0.015
Problem avoidance	6.13 (5.29)	4.74 (3.89)	0.30	2.07	0.004
Social withdrawal	8.15 (5.26)	8.30 (5.74)	−0.03	−0.18	0.861
**Second factor scales**					
Problem focused engagement	25.43 (8.29)	22.34 (8.84)	0.36	2.36	0.019
Emotion focused engagement	19.51 (9.27)	16.90 (10.02)	0.27	1.77	0.077
Problem focused disengagement	21.22 (6.98)	17.70 (6.58)	0.51	3.33	0.001
Emotion focused disengagement	17.47 (10.08)	17.75 (10.61)	−0.03	−0.18	0.862
**Third factor scales**					
Engagement	44.94 (14.70)	39.25 (16.64)	0.37	2.41	0.017
Disengagement	38.70 (14.57)	35.46 (15.02)	0.22	1.43	0.156

In order to determine which coping strategies are associated with CG risk, a multiple linear regression was performed. Scores of CG, as dimensional variables, were the dependent variable, and coping strategies were considered the independent variable. The results are presented in Table 4. Those coping strategies defined also as dimensional variables associated with CG were express emotions and social withdrawal.

**Table 4:** Multiple regression analysis of coping strategies associated with complicated grief scores.

	Unstandardized coefficients		Standardized coefficients			
	B	SE	Beta	*t*	Sig.	95% CI [LB UB]
Constant	1,82	4,63		0.39	0.695	[−7.31, 10.95]
Problem solving	0.302	0.26	0.09	1.18	0.240	[−0.20, 0.81]
Self-criticism	−0.043	0.18	0.06	0.79	0.815	[0.68, 1.62]
Express emotions	0.499	0.21	0.18	2.38	0.019	[0.09, 0.91]
Wishful thinking	0.179	0.27	0.06	0.66	0.509	[−0.35, 0.71]
Social support	0.058	0.24	0.02	0.24	0.809	[−0.42, 0.54]
Cognitive restructuring	−0.095	0.27	−0.03	−0.35	0.724	[−0.62, 0.43]
Problem avoidance	0.155	0.27	0.05	0.56	0.577	[−0.39, 0.70]
Social withdrawal	0.964	0.25	0.31	3.80	0.001	[0.46, 1.46]
	**R**	**R^2^**	**Adjusted R^2^**			
Model summary	0.400	0.160	0.124			

## Discussion

This study examined the relationship between coping strategies and CG in a sample of SUD patients. To our knowledge this is the first study to analyze coping and its relationship with two concurrent complex mental health conditions, CG and substance use disorder, in the same sample. This raises the question of whether loss among SUD patients may be associated with a different form of coping strategy than those observed in previously studied CG populations.

In general, the results supported previous research in which both CG and substance consumption are related to coping with potentially threatening situations.

As expected, the relationships observed between CG and dysfunctional coping patterns were stronger than engagement strategies in an SUD sample. Despite some correlations were weak and this indicates that there is minimal relationship between the variables, the tendency of the results were that problem focused disengagement and emotional focused disengagement were positively associated with CG. Specifically, wishful thinking and social withdrawal were the strategies most strongly linked to CG in all its facets. Wishful thinking is a cognitive strategy that implies another reality, formed by the beliefs and decision making based on what people might be pleased to imagine, instead of appealing to evidence, rationality, or reality. This was in line with the fact that, in some cases after a loss of a significant person, grief response can involve a lack of acceptance of the death and lead to complications in bereavement (Crunk et al., 2017; Nakajima, 2018).

Contrary to our expectations, and with previous evidence that avoidance is associated with negative outcomes among bereaved people (Bonanno, 2005) and more severity in CG symptoms (Shear et al., 2007), it is interesting to note that there was no relationship between problem avoidance and CG in the overall sample, except with the empty feelings subscale of the IGC. Considering more integrative models of coping, and according to some authors, it is important to point out that flexibility in coping is a mechanism to adapt to stressful life events (Park et al., 2015), and different coping strategies may be adaptive in different points in time or under different circumstances (Burton et al., 2012).

A remarkable difference was found in the CG SUD group in comparison with the adaptative grief SUD group. Problem and Emotional Disengagement identified CG group in a significative way. The strategies based on wishful thinking and self-critical judgments against oneself or self-blame can be an improper handling of the stressful situation in CG. In the same line, social withdrawal represented the other dysfunctional mechanism of the emotional disengagement in patients with SUD and CG. These results are consistent with those found by Schnider et al. (2007), the authors found that emotional avoidant coping was a significant predictor of CG in a college sample. The CG group also used more express emotions management as a functional management mechanism. These strategies are aimed at releasing emotions linked to stressful situations.

When we analyze the differences between groups classified according to the substance consumed, we found no differences in CG between the alcohol group and the cocaine and heroin group. Analyzing whether there are differences between patients for alcohol consumption (legal drugs) and for cocaine and heroin consumption (illegal drugs) is important because at the clinical level the therapeutic groups are often separated. In terms of coping, patients with a main diagnosis of alcohol dependence used more strategies, and higher than those related to wishful thinking, cognitive restructuring, and problem avoidance. The difference found in cognitive restructuring could be associated with cognitive impairments that are assumed to differ between substances (Bruijnen et al., 2019). There is evidence that, in opioid abuse, there exist impairments to the memory domain and to executive functioning, such as verbal fluency, inhibition, and decision making (Gruber et al., 2007). These functions are necessary in cognitive restructuring and problem resolution. Concerning chronic stimulant abuse, cognitive impairment is also present (Spronk et al., 2013).

Finally, our work concluded that social withdrawal and expression of emotion coping strategies were predictive variables of a CG dimensional factor. On the one hand, the active avoidance of contact with other people and, on the other, the need to release emotions were two of the resources that identified the presence of CG in people with SUD. According to previous research, CG tends to be associated with emotion-centered rather than problem-centered coping (Mancini and Bonano, 2019) and avoidance strategies are more related to courses with a worse prognosis (Feil and Hasking, 2008).

Overall, these results indicated that the co-occurrence of two serious health conditions (addiction and CG) was related to poorer management of stressful situations. Maladaptive coping, characterized by using disengagement strategies and an important lack of engagement strategies, was the main result found in this study.

The current research had some limitations. One of the most important is the cross-sectional design, which was an impediment to consider and justify the directionality (cause-effect) relationship between CG and coping in the SUD sample. Future longitudinal studies are necessary in order to establish a proper sequence of the events occurred. The second limitation was the size of our sample, as well as the use of a convenience sample which limit the generalization of our results. Another important limitation was that the presence of psychiatric disorders that could be associated with CG was not evaluated in the present study. Fourth, coping assessment was relied on a self-reporting measure (CSI). Finally, in this study, multiple comparisons correction was not made. Despite these limitations, the study provided significant new data related to the specificity of the sample. Future research should analyze the impact of other sociodemographic, personality, and clinical variables related to the coping strategies among SUD population. The period since the death of the significant person is another of the variables that should be analyzed in depth. Trajectories of CG can vary between subjects and numerous risk factors can be associated.

Our findings may contribute to a better understanding of the different variables involved in the grieving process among people with SUD. It is important to point out the clinical implications of addressing which coping strategies are associated with a better evolution of grief in people with addiction problems. Clinical benefits can be derived from these results. In one hand, training people to employ effective coping strategies in their lives could prevent different health and psychological problems. On the other hand, focusing therapy groups on identifying and raising awareness of less adaptive strategies used will have a very beneficial effect on the course of substance use disorder. More personalized interventions are important in the process of recovery and specific interventions should target those people with special clinical conditions. New treatment approaches for SUD individuals with CG should focus primarily on decreasing frequently occurring mechanisms, such as self-criticism, desiderative thinking, and social withdrawal, and increasing emotional and cognitive strategies linked to better management of stressful situations, such as problem solving, cognitive restructuring, and social support.

## Data Availability Statement

The raw data supporting the conclusions of this article will be made available by the authors, without undue reservation.

## Ethics Statement

The studies involving human participants were reviewed and approved by Institutional Ethics and Research Review Board of the Institut d’Assistència Sanitària (IAS). The patients/participants provided their written informed consent to participate in this study.

## Author Contributions

BC and LM conceived and designed the research. LM collected field data. BC wrote the manuscript. Both authors interpreted the results and approved the final manuscript.

### Conflict of Interest

The authors declare that the research was conducted in the absence of any commercial or financial relationships that could be construed as a potential conflict of interest.

## References

[ref1] Adan A. Antúnez J. M. Navarro J. F. (2017). Coping strategies related to treatment in substance use disorder patients with and without comorbid depression. Psychiatry Res. 251, 325–332. 10.1016/j.psychres.2017.02.035, PMID: 28237911

[ref2] Al-Gamal E. Alzayyat A. Ahmad M. (2016). Prevalence of internet addiction and its association with psychological distress and coping strategies among university students in Jordan. Pers. Psychiatr. Care. 52, 49–61. 10.1111/ppc.12102, PMID: 25639746

[ref3] Anderson K. G. Ramo D. E. Brown S. A. (2006). Lifestress, coping and comorbid youth: an examination of the stress-vulnerability model for substance relapse. J. Psychoactive Drugs 38, 255–262. 10.1080/02791072.2006.10399851, PMID: 17165368

[ref4] Boelen P. A. Prigerson H. G. (2007). The influence of symptoms of prolonged grief disorder, depression, and anxiety on quality of life among bereaved adults: a prospective study. Eur. Arch. Psychiatry Clin. Neurosci. 257, 444–452. 10.1007/s00406-007-0744-0, PMID: 17629728

[ref6] Bonanno G. A. (2005). Resilience in the face of potential trauma. Curr. Dir. Psychol. Sci. 14, 135–138. 10.1111/j.0963-7214.2005.00347.x

[ref7] Bonanno G. A. (2006). Is complicated grief a valid construct? Clin. Psychol. Sci. Pract. 13, 129–134. 10.1111/j.1468-2850.2006.00014.x

[ref8] Bruijnen C. Dijkstra B. Walvoort S. Markus W. Vandernagel J. Kessels R.. (2019). Prevalence of cognitive impairment in patients with substance use disorder. Drug Alcohol Rev. 38, 435–442. 10.1111/dar.12922, PMID: 30916448 PMC6593747

[ref9] Burton C. Yan O. Pat-Horenczyk R. Chan I. Ho S. Bonanno A. (2012). Coping flexibility and compolicated grief: a comparison of American and Chinese samples. Depress. Anxiety 29, 16–22. 10.1002/da.20888, PMID: 21898713 PMC3242921

[ref10] Cano F. J. Rodríguez L. García J. (2006). Adaptación española del Inventario de Estrategias de Afrontamiento. Actas Esp. Psiquiatr. 2, 29–39.

[ref11] Coriale G. Bilotta E. Leone L. Cosimi F. Porrari R. De Rosa F.. (2012). Avoidance coping strategies, alexithymia and alcohol abuse: a mediation analysis. Addict. Behav. 37, 1224–1229. 10.1016/j.addbeh.2012.05.018, PMID: 22727789

[ref12] Coyne J. C. Racioppo M. W. (2000). Never the twain shall meet?, Closing the gap between coping research and clinical intervention research. Am. Psychol. 55, 655–664. 10.1037//0003-066x.55.6.655, PMID: 10892208

[ref52] Crunk A. E. Burke L. A. Robinson E. (2017). Complicated grief: an evolving theoretical landscape. Couns. Dev. 95, 226–233. 10.1002/jcad.12134, PMID: 10892208

[ref13] Denny G. M. Lee L. J. (1984). Grief work with substance abusers. J Subst Abuse Treatment 1, 249–254. 10.1016/0740-5472(84)90003-5 6536768

[ref14] Feil J. Hasking P. (2008). The relationship between personality, coping strategies and alcohol use. Addict. Res. Theory 16, 526–537. 10.1080/16066350802025714

[ref15] Fujisawa D. Miyashita M. Nakajima S. Ito M. Kato M. Kim Y. (2010). Prevalence and determinants of complicated grief in general population. J. Affect. Disord. 127, 352–358. 10.1016/j.jad.2010.06.008, PMID: 20580096

[ref16] Furr S. R. Johnson W. D. Goodall C. S. (2015). Grief and recovery: the prevalence of grief and loss in substance abuse treatment. J. Addict. Offender Couns. 36, 43–56. 10.1002/j.2161-1874.2015.00034.x

[ref17] Gruber S. A. Silveri M. M. Yurgelun-Todd D. A. (2007). Neuropsychological consequences of opiate use. Neuropsychol. Rev. 17, 299–315. 10.1007/s11065-007-9041-y, PMID: 17690984

[ref18] Hasking P. A. Oei T. P. S. (2002). The differential role of alcohol expectancies, drinking refusal self-efficacy and coping resources in predicting alcohol consumption in community and clinical samples. Addict. Res. Theory 10, 465–494. 10.1080/1606635021000034049

[ref19] Hyman S. M. Hong K. A. Chaplin T. M. Dabre Z. Comegys A. D. Kimmerling A.. (2009). A stress-coping profile of opioid dependent individuals entering naltrexone treatment: a comparison with healthy controls. Psychol. Addict. Behav. 23, 613–619. 10.1037/a0017324, PMID: 20025367 PMC2802459

[ref20] Johnson V. Pandina R. J. (2000). Alcohol problems among a community sample: longitudinal influences of stress, coping and gender. Subst. Use Misuse 35, 669–686. 10.3109/10826080009148416, PMID: 10807151

[ref21] Kersting A. Brahler E. Glaesmer H. Wagner B. (2011). Prevalence of complicated grief in a representative population-based simple. J. Affect. Disord. 131, 339–343. 10.1016/j.jad.2010.11.032, PMID: 21216470

[ref53] Kersting A. Kroker K. Horstmann J. Ohrmann P. Baune B. Arolt V.. (2009). Complicated grief in patients with unipolar depression. J. Affect. Disord. 118, 201–204. 10.1016/j.jad.2009.01.033, PMID: 19268371

[ref22] Latham A. Prigerson H. (2004). Suicidality and bereavement: complicated grief as psychiatric disorder presenting greatest risk for suicidality. Suicide Life Threat. Behav. 34, 350–362. 10.1521/suli.34.4.350.53737, PMID: 15585457 PMC1459278

[ref54] Lazarus R. S. (1991). Emotion and adaptation. New York, NY: Oxford University Press.

[ref24] Limonero J. Lacasta M. García J. Maté J. Prigerson H. (2009). Adaptación al castellano del inventario de duelo complicado. Med. Pal. 16, 291–297.

[ref25] Lobb E. Janson K. Aoun S. Monterosso L. Halkett G. Davies A. (2010). Predictors of complicated grief: a systematic review of empirical studies. Death Stud. 34, 673–698. 10.1080/07481187.2010.496686, PMID: 24482845

[ref26] Mancini A. D. Bonano G. A. (2019). Predictors and parameters of resilience to loss: toward and individual differences model. J. Pers. 77, 1805–1832. 10.1111/j.1467-6494.2009.00601.x, PMID: 19807863 PMC4224188

[ref28] Masferrer L. Garre-Olmo J. Caparrós B. (2015). Risk of suicide: its occurrence and related variables among bereaved substance users. J Subst Use 21, 191–197. 10.3109/14659891.2014.998733

[ref27] Masferrer L. Garre-Olmo J. Caparrós B. (2017). Is complicated grief a risk factor for substance use? A comparison of substance-users and normative grievers. Addict. Res. Theory 25, 361–367. 10.1080/16066359.2017.1285912

[ref51] Nakajima S. (2018). Complicated grief: recent developments in diagnostic criteria and treatment. Philos. Trans. R. Soc. Lond. B. Biol. Sci. 373. 10.1098/rstb.2017.0273 PMC6053994 30012739

[ref29] Newson R. Boelen P. A. Hek K. Hofman A. Tiemeier H. (2011). The prevalence and characteristics of complicated grief in older adults. J. Affect. Disord. 132, 231–238. 10.1016/j.jad.2011.02.021, PMID: 21397336

[ref30] Pal H. R. Chavan B. S. (1996). Coping behaviours in recent abstinence attempt in opioid dependent subjects. Indian J. Psychiatry 38, 30–33. 21584114 PMC2970777

[ref31] Park M. Chang E. R. You S. (2015). Protective role of coping flexibility in PTSD and depressive symptoms following trauma. Pers. Individ. Differ. 82, 102–106. 10.1016/j.paid.2015.03.007

[ref32] Piper W. E. Ogrodniczuk J. S. Azim H. F. Weideman R. (2001). Prevalence of loss and complicated grief among psychiatric outpatients. Psychiatr. Serv. 52, 1069–1074. 10.1176/appi.ps.52.8.1069, PMID: 11474053

[ref100] Prigerson H. G. Ahmed I. Silverman G. K. Saxena A. K. Maclejewski P. K. Jacobs S. C. (2002). Rates and risks of complicated grief among psychiatric clinic patients in Karachi, Pakistan. Death Stud. 26, 781–792. 10.1080/07481180290106571 12440418

[ref33] Prigerson H. G. Frank E. Kasl S. V. Reynolds C. F. Anderson B. Zubenko G. S. (1995a). Complicated grief and bereavement related depression as distinct disorders: preliminary empirical validation in elderly bereaved spouses. Am. J. Psychiatry 152, 22–30. 10.1176/ajp.152.1.22 7802116

[ref34] Prigerson H. G. Maciejewski P. K. Reynolds C. F. Bierhals A. J. Newsom J. T. Fasiczka A. (1995b). Inventory of complicated grief: a scale to measure maladaptive symptoms of loss. Psychiatry Res. 59, 65–79. 8771222 10.1016/0165-1781(95)02757-2

[ref37] Schnider K. R. Elhai J. D. Gray M. J. (2007). Coping style use predicts posttraumatic stress and complicated grief symptom severity among college student reporting a traumatic loss. J. Couns. Psychol. 54, 344–350. 10.1037/0022-0167.54.3.344

[ref36] Shear K. Monk T. Houck P. Melhem N. Frank E. Reynolds C.. (2007). An attachment-based model of complicated grief including the role of avoidance. Eur. Arch. Psychiatry Clin. Neurosci. 257, 453–461. 10.1007/s00406-007-0745-z, PMID: 17629727 PMC2806638

[ref38] Simon N. M. Pollack M. H. Fischmann D. Perlman C. Muriel A. Moore C.. (2005). Complicated grief and its correlates in patients with bipolar disorder. J. Clin. Psychiatry 66, 1105–1110. 10.4088/jcp.v66n0903, PMID: 16187766

[ref39] Skinner E. A. Zimmer-Gembeck M. J. (2016). “Ways and families of coping as adaptive processes” in The development of coping. ed. Fisker S. T. (Cham: Springer).

[ref40] Spronk D. B. van Wel J. H. P. Ramaekers J. G. Verkes R. J. (2013). Characterizing the cognitive effects of cocaine: a comprehensive review. Neurosci. Biobehav. Rev. 37, 1838–1859. 10.1016/j.neubiorev.2013.07.003, PMID: 23876288

[ref41] Stroebe M. Schut H. Stroebe W. (2007). Health outcomes of bereavement. Lancet 370, 1960–1973. 10.1016/S0140-6736(07)61816-9, PMID: 18068517

[ref42] Tariq M. Jameel R. (2020). Stigmatization, self-criticism and coping strategies of individual with subtance abuse. Res. Soc. Work. Pract. 8, 1–10.

[ref43] Taylor S. E. Stanton A. L. (2007). Coping resources, coping processes, and mental health. Annu. Rev. Clin. Psychol. 3, 377–401. 10.1146/annurev.clinpsy.3.022806.091520, PMID: 17716061

[ref44] Tobin D. L. Holroyd K. A. Reynolds R. V. Wigal J. K. (1989). The hierarchical factor structure of coping strategies inventory. Cognit Ther Res 13, 343–361.

[ref45] Walker R. Stephens R. S. (2014). Protective behavioral strategies mediate problem-focused coping and alcohol use in college students. Psychol. Addict. Behav. 39, 1033–1037. 10.1016/j.addbeh.2014.02.006, PMID: 24629479

[ref46] Wills T. A. Hirky A. E. (1996). “Coping and substance abuse: a theoretical model and review of the evidence” in Handbook of coping: Theory research and applications. eds. Zeichnec M. Eudler N. S. (New York: Wiley), 279–302.

[ref47] Wittouck C. Van Autreve S. De Jaegere E. Portzky G. van Heeringen K. (2011). The prevention and treatment of complicated grief: a meta analysis. Clin. Psychol. Rev. 31, 69–78. 10.1016/j.cpr.2010.09.005, PMID: 21130937

[ref49] Zisook S. Lyons L. (1990). Bereavement and unresolved grief in psychiatric outpatients. Omega 20, 307–322. 10.2190/A4U5-T46H-B0HJ-DBRQ

[ref48] Zisook S. Shuchter S. Schuckit M. (1985). Factors in the persistence of unresolved grief among psychiatric outpatients. Psychosomatics 26, 497–503. 4011815 10.1016/S0033-3182(85)72830-7

[ref50] Zuckoff A. Shear K. Frank E. Daley D. Seligman K. Silowash R. (2006). Treating complicated grief and substance use disorders: a pilot study. J. Subst. Abus. Treat. 30, 205–211. 10.1016/j.jsat.2005.12.001, PMID: 16616164

